# *In planta* Transformed Cumin (*Cuminum cyminum* L.) Plants, Overexpressing the *SbNHX1* Gene Showed Enhanced Salt Endurance

**DOI:** 10.1371/journal.pone.0159349

**Published:** 2016-07-13

**Authors:** Sonika Pandey, Manish Kumar Patel, Avinash Mishra, Bhavanath Jha

**Affiliations:** 1 Marine Biotechnology and Ecology Division, CSIR-Central Salt and Marine Chemicals Research Institute, G. B. Marg, Bhavnagar, Gujarat, 364002, India; 2 Academy of Scientific and Innovative Research, CSIR, New Delhi, India; Hainan University, CHINA

## Abstract

Cumin is an annual, herbaceous, medicinal, aromatic, spice glycophyte that contains diverse applications as a food and flavoring additive, and therapeutic agents. An efficient, less time consuming, *Agrobacterium-*mediated, a tissue culture-independent *in planta* genetic transformation method was established for the first time using cumin seeds. The *SbNHX1* gene, cloned from an extreme halophyte *Salicornia brachiata* was transformed in cumin using optimized *in planta* transformation method. The *SbNHX1* gene encodes a vacuolar Na^+^/H^+^ antiporter and is involved in the compartmentalization of excess Na^+^ ions into the vacuole and maintenance of ion homeostasis Transgenic cumin plants were confirmed by PCR using gene (*SbNHX1*, *uidA* and *hptII*) specific primers. The single gene integration event and overexpression of the gene were confirmed by Southern hybridization and competitive RT-PCR, respectively. Transgenic lines L3 and L13 showed high expression of the *SbNHX1* gene compared to L6 whereas moderate expression was detected in L5 and L10 transgenic lines. Transgenic lines (L3, L5, L10 and L13), overexpressing the *SbNHX1* gene, showed higher photosynthetic pigments (chlorophyll a, b and carotenoid), and lower electrolytic leakage, lipid peroxidation (MDA content) and proline content as compared to wild type plants under salinity stress. Though transgenic lines were also affected by salinity stress but performed better compared to WT plants. The ectopic expression of the *SbNHX1* gene confirmed enhanced salinity stress tolerance in cumin as compared to wild type plants under stress condition. The present study is the first report of engineering salt tolerance in cumin, so far and the plant may be utilized for the cultivation in saline areas.

## Introduction

Cumin **(***Cuminum cyminum* L.) is an aromatic medicinal herb; dried seeds are consumed as spices, and the plant is well documented with various functional and nutritional properties in the scientific literature. Spices are an important constituent of human nutrition and non-leafy plant parts such as seed, fruit and flower are not only used for flavor enhancement but also as food preservatives [1–2]. Cumin is most widely used plant of Apiaceae family because of multifunctional role with therapeutic applications [3–5] such as antibacterial, antioxidant[6], antidiabetic and also a good source of vitamin B-complex and minerals like iron, calcium and zinc [7]. The active constituent, cuminaldehyde is present in essential oil of cumin, which possesses anti-inflammatory activity [3, 8], anti-aflatoxigenic property and anti-fibrillation activity [9].

The plant attains a height of 30–40 cm, thrives well in tropical regions because of the fibrous root, and also growth period coincides with winter and springs rainfall that provides drought tolerance ability [6–7, 10]. However, productivity and seed yield of cumin is highly susceptible to soil salinization *i*.*e*. salinity stress which is irresistible process in semi-arid regions due to natural occurrence of high amount of soluble salt [11–12], uneven rainfall distribution and poor quality water for irrigational purposes leading to excess salt deposition in the root zone of growing plants [7, 13–14]. Previous studies conducted on cumin cultivation have summarized that vegetative stage and flower setting stage of cumin plant is most sensitive showing tolerance level up to 5 ds/m in salt affected land [15]. The germination of seed, seedling growth and other metabolic activities of this aromatic plant is severely affected by salinity stress which further decreases umbel formation and seed harvest index causing yield and productivity loss of cumin seeds [5, 15–16]. In addition, the present scenario of agricultural land highly threatens to productivity loss, as about 20% (62 million hectares) of irrigated land has already subjected to salt-induced degradation [17].

Salinity tolerance is multi-faceted trait governed by a plethora of mechanism that varies with plant development stage or environmental factors that are highly dynamic in nature [18]. Therefore, incorporation of salinity tolerance in domesticated crops by transferring salt tolerant gene is one of the major challenges for plant breeders. Although cumin is an economic cash crop, limited efforts have been made for varietal development [19], also traditional breeding approaches have not proven beneficial due to narrow germplasm range [20] and small floral size further creates difficulties in emasculation and hybridization [21–22]. Moreover limited reports are available on the intervention of biotechnological tools in cumin. Earlier, we have established an efficient microprojectile bombardment- and *Agrobacterium*-mediated genetic transformation of cumin for the first time using embryos as explants [7, 23].

Advancement in the transformation techniques have revolutionized the methodology for crop improvement, supports functional validation of genes and promoters [24–30] and a number of genetically modified crops have been developed containing the gene of interest through transgenic approaches [31–34]. Routinely for producing transgenic plants, the procedure comprises of transferring the desired gene into a highly totipotent explant (callus, embryo, leaf, shoot apex etc.) via *Agrobacterium* or particle bombardment and then following tissue culture based regeneration protocol for complete plant development [23, 35]. Although the *in vitro* method of plant regeneration-transformation is applicable to variety plants species [36–38] but the method is laborious and not promising because of the requirement of sterile conditions [39] and also somaclonal variations or morphological abnormalities as a result of the long phase of regeneration [40–42]. Also, most of the plant species are highly recalcitrant to *in vitro* regeneration [39, 43]. To overcome the constraints mentioned above, the *in vivo* method of *Agrobacterium*-mediated transformation, known as *in planta* are gaining more importance.

High salinity disturbs the ionic homeostasis within the cell by inhibiting the normal physio-biochemical metabolism promoting plant senescence [44]. Therefore, selective ion uptake followed by compartmentalization is a very useful strategy to keep away toxic ions (Na^+^) from cellular machinery for normal growth or to sustain growth under saline conditions [45]. Na^+^/H^+^ antiporters (NHXs) are an essential cation/proton antiporters localized universally to the plasma membrane and endomembranes of many cells ranging from prokaryotes to eukaryotes (higher plants and animals) [46]. The importance of vacuolar Na^+^ compartmentalization in plant salt tolerance has been demonstrated in transgenic plants by overexpressing the *NHX1* gene. Previously, the potentiality of *SbNHX1* gene isolated from *Salicornia brachiata* as salt tolerant has been checked in *Jatropha curcas* [31] and *Ricinus communis* [33] through transgenic approach. *Salicornia* is an extreme halophyte, possessed unique characteristics [47–49] and it was observed that overexpression of the *SbNHX1* in transgenic plants modulated the physiological and biochemical response that improved growth and enhanced salt tolerance than wild type plant [31, 33].

Till date, a number of successful *in planta* transformation was performed with *Agrobacterium* using germinated seeding of plants like *Raphanus sativus*, *Saccharum officinarum*, *Oryza sativa*, *Solanum melongena* and *Brassica napus* [39, 50–52]. The germinating seeds are readily available throughout the year and easy to handle, and also plants obtained through seed transformation have been found to be truly transformed [50, 52].In the present study, an *in planta* transformation method for *C*. *cyminum* has been carried out using the gene *SbNHX1* to enhance the salt tolerance. The *Agrobacterium* strain EHA105 containing the gene construct pCAMBIA1301-*SbNHXI* was transferred assisted by vacuum infiltration of germinated seeds to develop transgenic cumin plants that can withstand with salinity stress and can be utilized for cultivation in the salt affected area.

## Materials and Methods

### Plant material and selection of basal media

Healthy and uniform mature cumin seeds of a local cultivar (GC-3) were rinsed with water and treated with 2% (v/v) NaOCl disinfectant for 10 minutes. Seeds were washed five to six times with sterile water, kept soaked in distilled water at 4°C for overnight to make seeds fully turgid. Four different basal media *viz* water agar (distilled water solidified with agar 0.8% w/v), B5 (Gamborg’s B5 basal medium including macronutrients, micronutrients and iron source + sucrose 3% w/v, solidified with agar 0.8% w/v), half MS (half strength Murashige and Skoogs medium with macronutrients, micronutrients and iron source + sucrose 3% w/v, solidified with agar 0.8% w/v) and MS (Murashige and Skoogs medium with macronutrients, micronutrients and iron source + sucrose 3% w/v, solidified with agar 0.8% w/v) were evaluated for the seed germination. The plant basal media was supplemented with sucrose (3% w/v), solidifying agent agar (0.8% w/v) and the pH was adjusted to 5.8 by adding 1 N NaOH or 1 N HCl before autoclaving. The surface sterilized seeds were inoculated in petri dishes containing solidified plant growth media (water agar, B5, half MS or MS basal) overlaid with sterilized filter paper (Whatman No. 1). Cultures were incubated in dark at 21°C for seven days, later on, transferred under 16/8 h photoperiod for one week at a light intensity of 35 μmol m^-2^ s^-1^ at 24 ± 2°C under controlled culture conditions [7, 23]. Seeds were germinated up to the emergence of the cotyledonary leaf.

### *In planta* transformation method

*Agrobacterium tumefaciens* (EHA105 strain) harboring the gene construct pCAMBIA1301-*SbNHX1* (31, 33) was used to perform *in planta* seed transformation. *Agrobacterium* culture was grown in 50 mL of LB broth supplemented with 50 mgL^−1^ kanamycin and 25 mgL^−1^ rifampicin at 28°C till absorbance of the bacterial culture reached to OD_600_ = 0.6 [23]. The cells were harvested by centrifugation at 2700 x g for 10 min, and the pellet was dissolved in equal volume of infiltration medium (½ MS salt; 300 μM Acetosyringone, AS and pH 5.7).

Mature surface sterilized seeds of cumin was used to optimize the various parameters that influence the *in planta* seed transformation including duration of pre-culturing of seed and vacuum infiltration. The sterilized pre-soaked seeds were pre-cultured for 5, 7, 9 and 11 days in petri dishes containing 50 mL solidified MS medium overlaid with filter paper (Whatman No. 1) in dark conditions at 21°C. The germinated seeds with emerged plumule were wounded by syringe needle dipped in *Agrobacterium* suspension at the apical meristem of the axis and the inter-cotyledonary region. After the injury, germinating seeds were inoculated with 100 mL infiltration medium (½ MS containing pellets of *Agrobacterium* + 300 μM AS; pH 5.7) and subjected to vacuum infiltration for 10 min at 250 mm of Hg. After vacuum treatment, cumin seeds were taken out from the infiltration medium and blotted dry on sterile tissue paper to get rid of the excess of *Agrobacterium* suspension. After that, seeds were transferred to petri dishes containing MS medium and incubated (co-cultivation) for 72 h in the dark at 23°C. Co-cultivated seeds were washed five times with sterile liquid ½ MS basal solution containing 300 mgL^−1^ cefotaxime, blot dried on sterile tissue paper, and transferred to soil for plant development. Transformed seedlings were grown in a growth chamber for further development at 24 ± 2°C under a 16/8 h photoperiod. Approximately after 4-weeks, flower bud initiation started and flowers were opened completely in the next two-weeks. At this stage, ear-bud was used on fully opened flowers for cross pollination. Plants were further grown till seed maturation and harvesting. The number of surviving seedlings were recorded and subjected to transgene confirmation.

### Histochemical GUS assay of *in planta* transformants

The expression of *uidA* (*gus*) gene was confirmed histochemically by dipping randomly selected germinating seedlings in the X-Gluc buffer (GUS assay kit, Sigma, USA) followed by incubating at 37°C for 12 h. Subsequently plant material was washed with 70% ethanol to remove the chlorophyll content and blue spots were examined under binocular stereomicroscope (Olympus, Japan).

### Molecular analysis of *in planta* transformants

Healthy leaves were collected from samples (wild type, WT; control/non transformed and transformed plants), genomic DNA was extracted, quantified and subjected to PCR amplification using genes specific primers [31, 33]. Southern hybridization was performed to determine copy number and stable integration of candidate gene. About 30 μg of purified genomic DNA (from WT and transformants) were digested with *Hind* III enzyme for 24 h at 37°C, separated on a 0.7% agarose gel and transferred to Hybond N^+^ membrane (Amersham Pharmacia, UK) by the capillary method using alkaline transfer buffer (0.4 N NaOH, 1 M NaCl). The gene construct pCAMBIA1301-*SbNHX1* and DNA from non-transformed plants were used as a positive and negative control, respectively. The membrane was neutralized with neutralization buffer (0.5 M Tris-Cl of pH 7.2 with 1 M NaCl), air-dried and DNA binding was fixed by UV cross-linking, using 56 mJ cm^−2^ energy for 1 min in a UVC 500 cross-linker (Amersham Biosciences, UK). After that blot had been hybridized with PCR-generated, DIG-11-dUTP labeled *SbNHX1* gene probe following PCR DIG probe synthesis kit (Roche, Germany). The hybridized membrane was detected by using CDP-Star chemiluminescent as a substrate, following the manufacturer’s instructions (Roche, Germany). The X- ray film (Eastman Kodak, USA) was processed to visualize the signals.

The overexpression of *SbNHX1* gene in transgenic plants was studied by semi-quantitative RT-PCR. Total RNA from leaves (WT and transgenic lines; L3, L5, L6, L10 and L13) was isolated using RNeasy plant mini kit (Qiagen, Germany) according to manufacturer’s protocol. The cDNA was synthesized using ImProm-II Reverse Transcription System kit (Promega, USA). Semi-quantitative RT-PCR analysis was performed using cDNA as a template, *SbNHX1* gene as a target and *actin* gene as an internal control.

### Physio-biochemical analysis of *in planta* transformants for salt tolerance

To determine the salt tolerance ability of transformed plants, different physiological and biochemical studies were carried out. For this, plants grown in plastic pots containing garden soil were irrigated with NaCl (200 mM) at regular two days interval. The effect of salt was observed after 15 days, and leaves samples were harvested from treated and untreated plants (WT and transgenic lines, L3, L5, L10 and L13) for different physio-biochemical analyses (electrolyte leakage, proline, malondialdehyde and chlorophyll).

### Estimation of electrolyte leakage, proline content, lipid peroxidation and photosynthetic pigments of *in planta* transformants under salt stress condition

The electrolyte leakage (EL) was estimated to determine salinity induced damage in leaves of transformed cumin and compared with control plants [44]. Leaves sample (WT and transgenic lines L3, L5, L10 and L13) were collected, washed to remove surface-adhered electrolytes, immersed in deionized water (10 mL), and incubated at 25°C for 24 h. The initial electrical conductivity (EC) of the solution (L_t_) was measured using conductivity meter (SevenEasy, Mettler Toledo AG 8603, Switzerland). After that leaf samples were boiled at 99°C for 20 min, cooled to room temperature, final electrical conductivity (L_0_) of the solution was measured, and the electrolyte leakage was determined.

The change in free proline content under salinity stress was monitored by a method described by Bates et al. [53]. The extract was prepared by homogenizing 20 mg leaves samples (WT and transgenic lines L3, L5, L10 and L13) in 3% sulphosalicylic acid, centrifuged at 10,000 rpm for 15 min at 4°C and supernatant was collected. Supernatant (1 mL) was mixed with acid ninhydrin (1 mL) and glacial acetic acid (1 mL) and incubated for 1 h in water bath at 98°C. After cooling on ice cold water, 2 ml toluene was added followed by vortexing for 15 sec. The upper phase was collected, and total proline was calculated by the standard curve, prepared against the known concentration of proline measured at 520 nm absorbance [53].

The MDA content was estimated for quantifying the lipid peroxidation in treated and untreated leaves samples (WT and transgenic lines L3, L5, L10 and L13) under salinity stress following the method as described by Hodges et al. [54]. Leaves samples (20 mg) were ground and extracted with trichloroacetic acid reagent (TCA; 2 mL, 0.1% w/v). Reaction samples were of two types; 1^st^ set contained TBA (0.65% w/v TBA prepared in TCA 20% w/v), and 2^nd^ set of the experiment included 20% w/v TCA (TBA was excluded) only. The plant extract (0.5 mL) was mixed with 2 mL of TBA with TCA or only TCA. Both reaction mixtures were incubated in water bath at 95°C for 30 min and later on cooled in ice water to stop the reaction. Supernatant was collected after centrifugation at 10,000 rpm for 5 min and finally absorbance was recorded at 440 nm, 532 nm and 600 nm, and MDA content was measured [54].

Fresh leaves of untreated and treated plants (WT and transgenic lines L3, L5, L10 and L13) were used for estimation of various photosynthetic pigments. Samples (20 mg) were extracted in 100% N, N-dimethylformamide (DMF) at 4°C and different photosynthetic pigment contents such as Chl a, Chl b, total Chl (a+b) and carotenoid were estimated using absorbance recorded at 665, 647 and 461 nm [55–56].

### Statistical analysis

All quantitative data, recorded from three replicates, were subjected to determine the significance of difference by analysis of variance (ANOVA) and graphs represent mean value ± SE followed by different letters are significantly different at *P*<0.05.

## Results

### Selection of basal media

**Fig 1:**
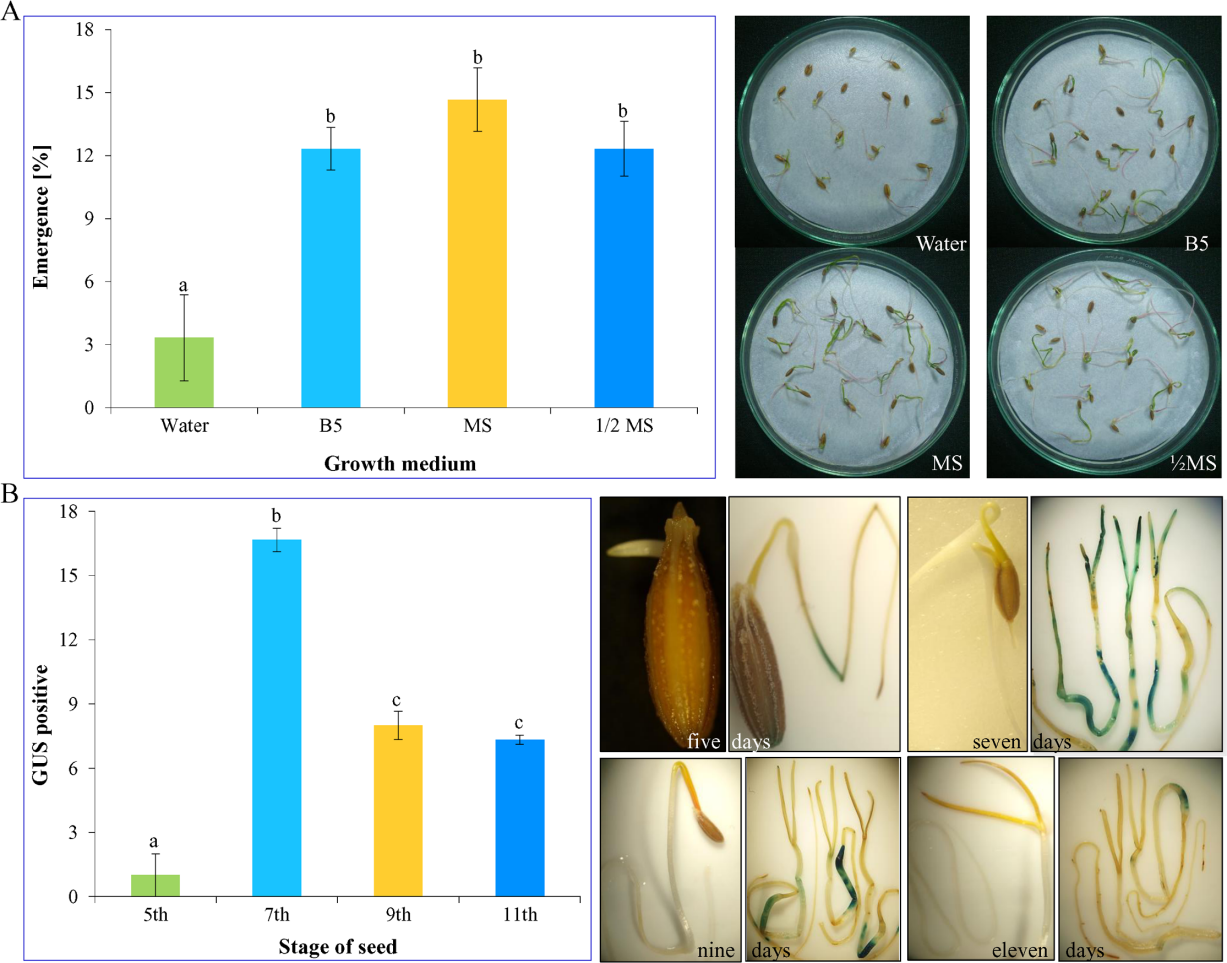
Effect of basal media and pre-culturing. Effect of different basal media on seed emergence (A). Seeds germinated on water, B5, ½ MS and MS. Effect of the stage of pre-cultured seeds on transformation frequency (B). Seeds were germinated and pre-cultured for 5, 7, 9 and 11 days. Graphs represent mean value ± SE followed by different letters are significantly different at *P*<0.05.

The pre-culturing of seeds was prerequisite for *in planta* transformation and for this, the effect of different basal media (water agar, B5, MS and ½ MS) on the emergence of cotyledonary leaf from seeds of cumin was evaluated, and growth response was recorded after ten days (**Fig 1**). Seeds inoculated on MS basal medium showed the best response towards germination as compared to other media (water agar, B5 and ½ MS) used in the study. The emergence percentage of the cotyledonary leaf was 4.4-fold higher in MS as compared to water agar. Though, B5 and ½ MS media showed similar response towards percent emergence which was about 3.7-fold higher than water agar medium but was lower than that of MS medium.

### Influence of pre-culturing of seed on transformation efficiency

The seeds were pre-cultured on MS basal medium for the different period (5, 7, 9 and 11 days) before infection with *Agrobacterium* and transformation efficacy was monitored by analyzing blue foci of the *uidA* (*gus*) gene expression (**Fig 1**). The GUS foci were found maximum in 7 days pre-culturing condition (*P*<0.05) followed by 9 to 11 days pre-culturing. The optimal pre-culture period was determined as seven days which showed highest *gus* expression that was 17-folds higher to 5 days pre-culturing. Furthermore, pre-cultured seeds of longer duration (9 and 11 days) showed two-fold lower response as compared to 7 days.

### Effect of vacuum infiltration

**Fig 2:**
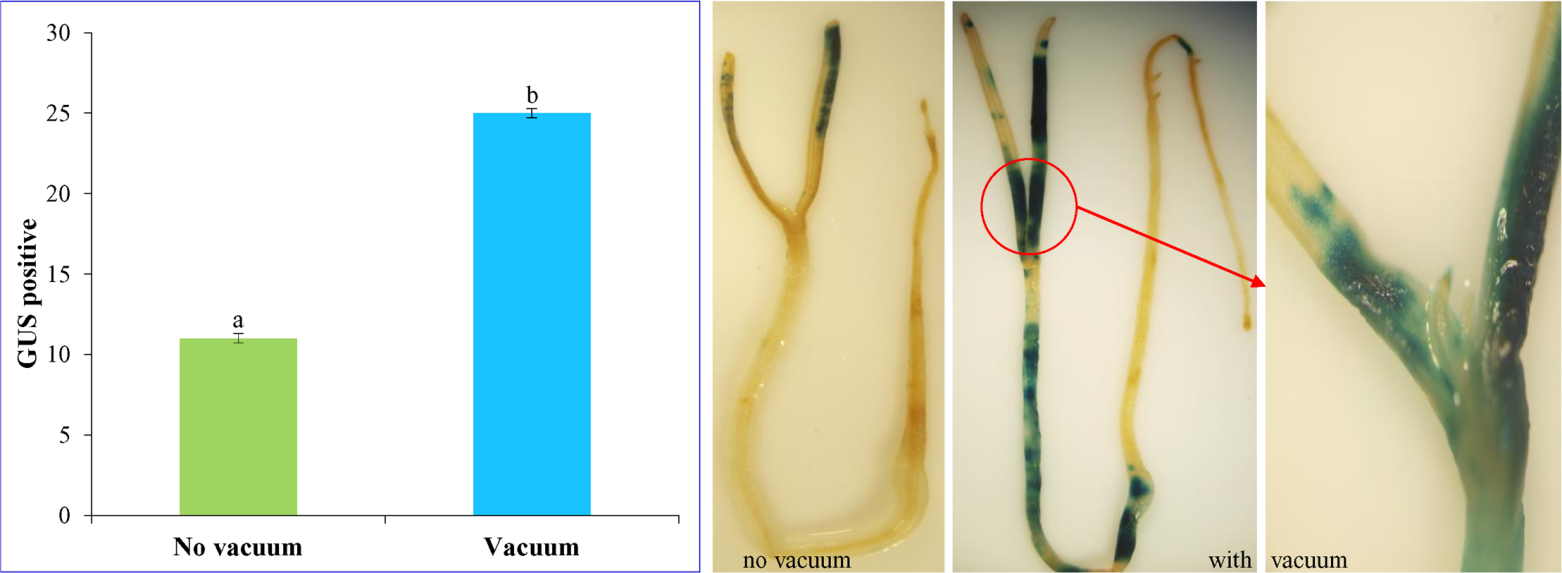
Effect of vacuum infiltration on transformation efficiency. The graph represents mean value ± SE followed by different letters are significantly different at *P*<0.05.

Seven days pre-cultured seeds were subjected to vacuum infiltration with *Agrobacterium* suspension for 10 min and monitored by *gus* expression (**Fig 2**). A radical increase in the efficiency of transformation was detected compared to control in which no vacuum-infiltration was applied. The mean number of *gus* positive was about 2.2 times higher in vacuum in-filtered as compared to no vacuum treatment. The intense blue spots were detected over the surface of the infiltered seedlings covering a major portion of the cotyledonary leaf, hypocotyl, and radicle. Thus, application of vacuum was found to be optimum for increasing the transformation frequency.

### *In planta* transformation and development of transgenic plants

**Fig 3:**
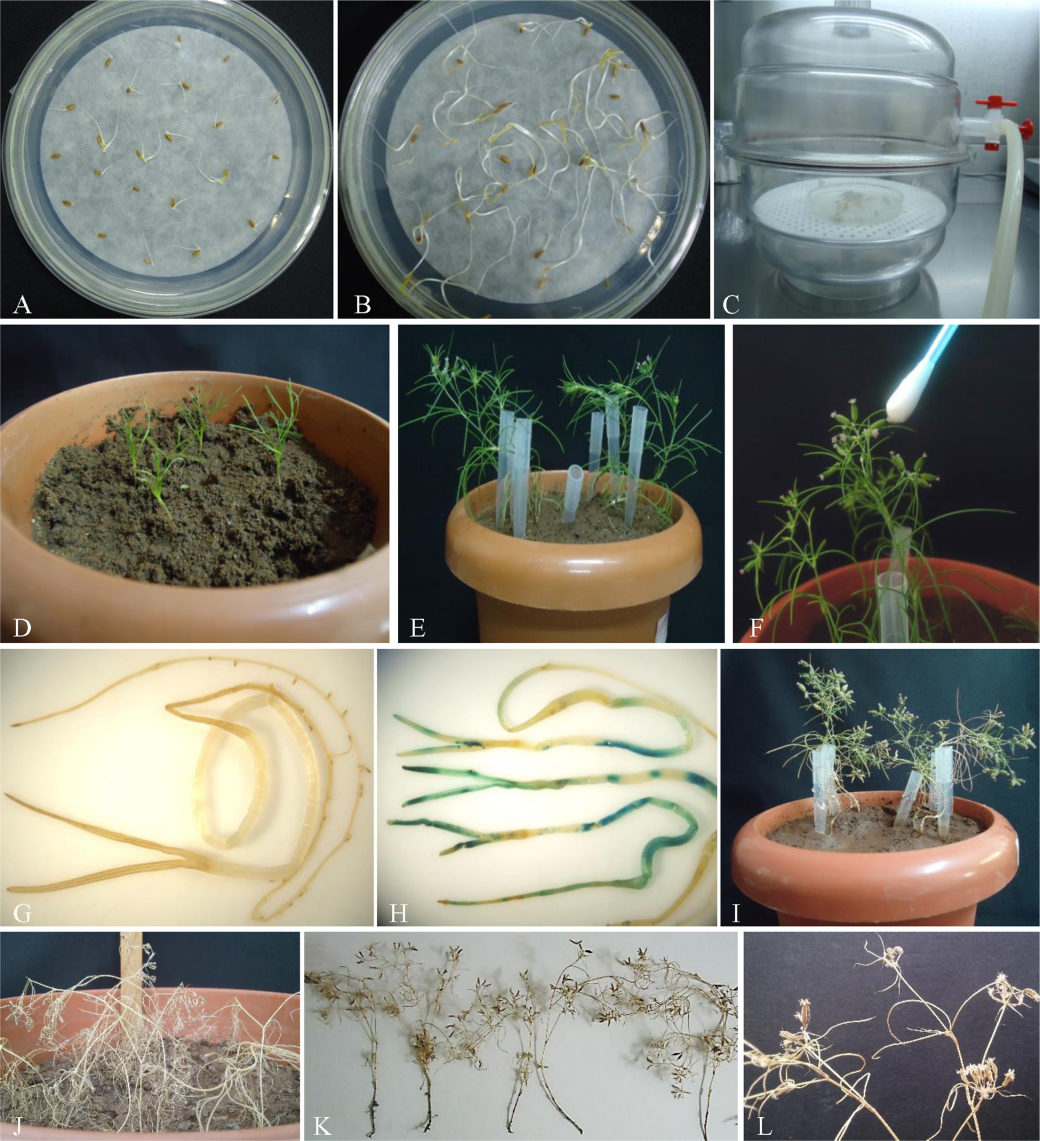
*In planta* transformation of cumin seeds. Seven days pre-cultured germinated seeds (A) were infected (co-cultivated) with *Agrobacterium* (B) followed by vacuum infiltration in a desiccator (C) and then transferred to the plastic pot for further growth (D). Putative transformants showed initiation of flower bud formation (E) and cross pollination by using ear bud (F). Randomly selected non transformed (control) seedling (G) and putatively transformed seedlings (H) showed histochemical GUS spots. Putative transgenic plants are grown further for seed setting and maturation (I-L).

**Fig 4:**
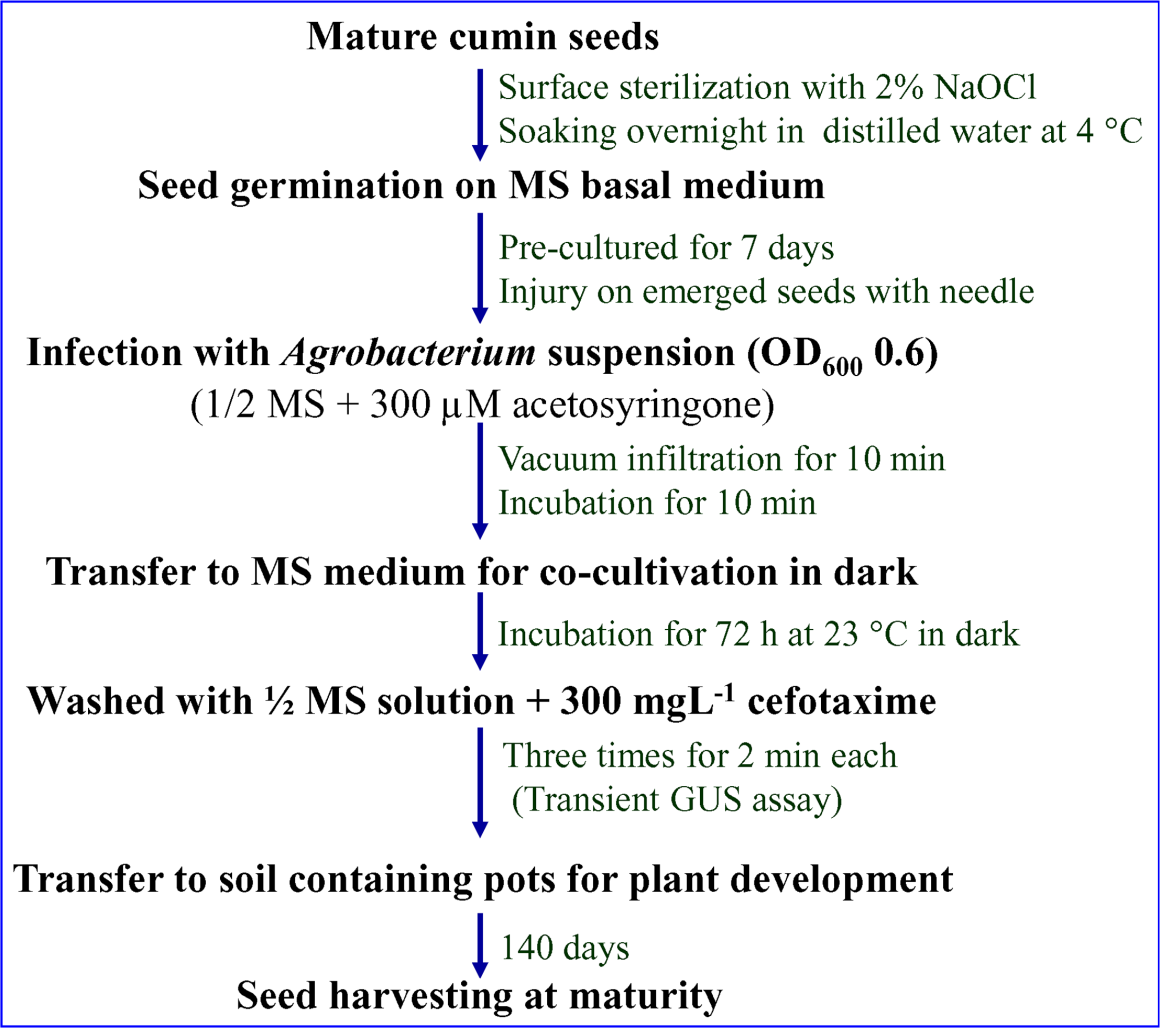
Schematic representation of optimized *in planta* transformation protocol used for *Agrobacterium*-mediated genetic transformation of cumin seeds.

*In planta* transformation of pre-incubated cumin seeds (on MS basal medium for 7 days) was performed using *Agrobacterium* suspension (of OD_600_ = 0.6) supplemented with 300 μM AS followed by 10 min vacuum infiltration (**Fig 3**). Out of the total 200 seeds subjected to *in planta* transformation, 17 plants survived and attained maturity (i.e. completed their life cycle). Thus, survival frequency was found to be 8.5%. However only 11 plants were able to set seed normally, and next generation seeds were obtained. The complete schematic representation of optimized *in planta* method for transformation in cumin seeds mediated by *A*. *tumefaciens* is given as **Fig 4.**

### Molecular analyses of *in planta* transformants

**Fig 5:**
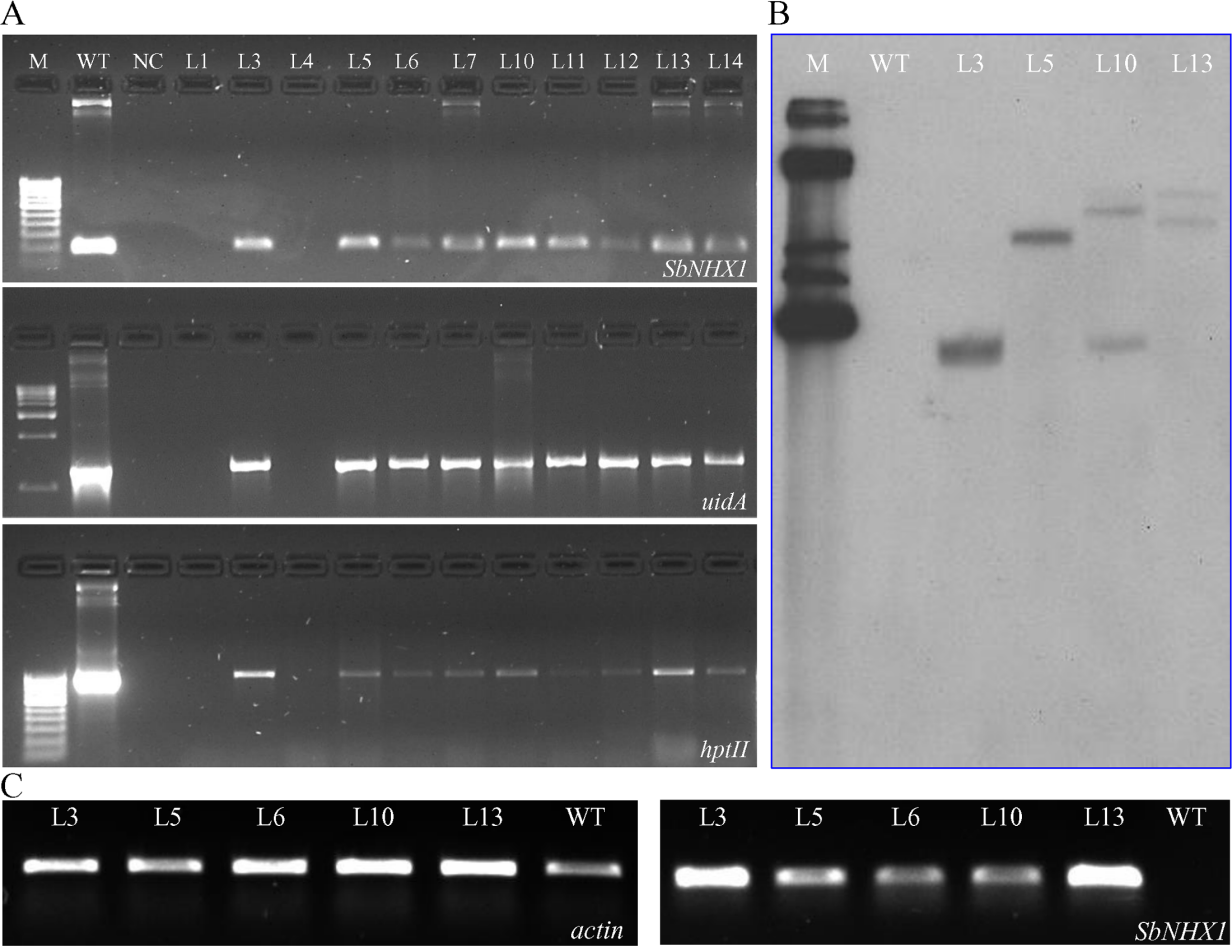
Molecular confirmation of *in planta* transformed transgenic lines. PCR confirmation of putative transgenic lines using *SbNHX1*, *gus* gene and *hptII* genes (A); Southern blot analysis of randomly selected *in planta* transgenic plants (B) and overexpression analysis of the *SbNHX1* gene in transgenic plants, analyzed by semi-quantitative RT-PCR (C). Lane M: Molecular weight marker ladder, lane PC: positive control, lane WT: wild type plant (negative control i.e. non-transformed plant) and lanes L: putative transgenic lines.

The genomic DNA was used as a template for PCR reaction for confirmation of gene integration into the plants of cumin. PCR amplification of genomic DNA isolated from cumin T_0_ transformants showed the presence of the *SbNHX1*, reporter gene *gus* and selectable marker *hptII* of expected bands of sizes 172 bp (internal fragment), 1280 bp and 963 bp respectively (**Fig 5**). A total of 11 transformed T_0_ plants obtained by *in planta* transformation were screened, out of which nine plants were found to be positive.

The Southern blot analysis of four randomly selected transformed T_0_ plants obtained by *in planta* method was performed to determine the copy number of *SbNHX1* in the genome of cumin (**Fig 5**). It was found that *SbNHX1* probe hybridized to a single fragment in transgenic lines L3 and L5 whereas multiple hybridizations were observed in L10 and L13. Thus, Southern blot analysis revealed the presence of single copy insertion in L3 and L5 and double copy gene insertion in L10 and L13 transgenic lines (**Fig 5**). The WT plants did not show any hybridization signal with the probe.

The overexpression of the *SbNHX1* gene was confirmed by competitive reverse transcriptase PCR (RT-PCR) (**Fig 5**). Transgenic lines (L3 and L13) showed the highest expression of the gene; moderate expression was detected in L5 and L10 transgenic lines while lowest expression was found in L6. Transgenic lines L3, L5, L10 and L13 were selected further for salt stress assay including physio-biochemical responses under salinity stress.

### Physio-biochemical analysis of *in planta* transformants

**Fig 6:**
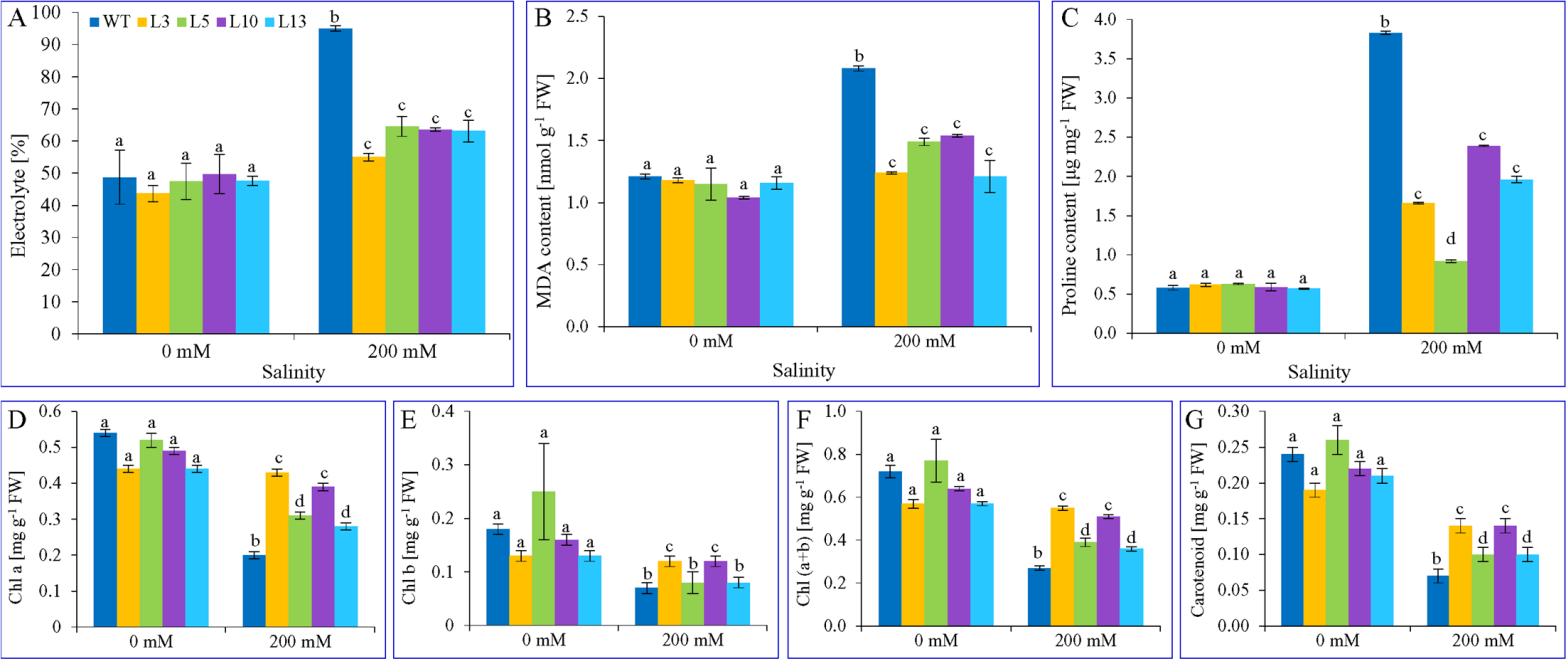
Bio-physiology of transgenic lines under salinity stress. Estimation of electrolyte leakage (A), lipid peroxidation (B), proline (C), chlorophyll a (D), chlorophyll b (E), total chlorophyll (F) and carotenoid contents (G) of WT and transgenic plants. Graphs represent mean value ± SE followed by different letters are significantly different at *P*<0.05.

Transgenic lines along with WT were treated with 200 mM NaCl salt solution for 15 days in soil at every two days interval. The effect of salinity stress was observed after 15 days. Leaves were harvested and used for measuring the level of electrolyte leakage, MDA, proline and photosynthetic pigments during salinity stress (**Fig 6**).

No significant difference (*P*<0.05) in the electrolyte leakage, proline content, and lipid peroxidation was found in WT and transgenic plants under control condition (0 mM NaCl). However under 200 mM NaCl stress condition, the leakage of electrolyte in WT increased by two-folds than that of control. The electrolyte leakage also increased for transgenic lines, but it was found to be significantly (*P*<0.05) lower compared to WT plants under stress condition (**Fig 6A**). The level of MDA was 1.7-folds higher in WT compared to control under stress condition (200 mM NaCl). In contrast, transgenic lines showed lower MDA accumulation to that of WT plants under 200 mM NaCl stress (**Fig 6B**). Similarly, the transgenic lines were found to accumulate less proline content as compared to WT, and a significant difference was observed under salt stress (**Fig 6C**).

In leaves of WT and transgenic plants, the chlorophyll a content showed no significant difference in the control condition (0 mM NaCl). The WT showed 63% reduction in chlorophyll a content under salt stress (200 mM NaCl) compared to control (**Fig 6D**). On the other side, the level of chlorophyll a was reduced about 2.2, 43, 21 and 37% in transgenic lines L3, L5, L10 and L13 respectively under salt stress (200 mM NaCl). In the case of chlorophyll b, WT plants showed 62% reduction in salt stress compared to control (**Fig 6E**). Transgenic lines were found to tolerate salinity stress by showing lower deterioration of chlorophyll under stress (200 mM NaCl). The percentage of total chlorophyll (a+b) in WT was decreased up to 62.5% under stress compared to control (**Fig 6F**). However transgenic lines L3, L5, L10 and L13 showed 3.6, 50, 21 and 37% reduction in total chlorophyll under NaCl stress (200 mM). While estimating the carotenoid content, WT plants showed approximately 70% reduction in stress (200 mM NaCl) compared to control (0 mM NaCl) condition (**Fig 6G**). On the contrary, the carotenoid content was decreased by 26, 38, 36 and 32% respectively in leaves of transgenic plants L3, L5, L10 and L11 treated with salt (200 mM NaCl). Overall, transgenic lines (L3, L5, L10 and L11) performed better compared to WT plants under salinity stress.

## Discussion

Salinity imposes a detrimental effect on the plant's life, and most of the plants, known as glycophytes cannot survive under saline condition. Tolerance to the salinity stress can be of utmost importance to provide enough food to the growing world population. Antiporters are a key determinant for maintaining cellular ion homeostasis within the plant cell thus averting the toxic effects of accumulated salt by compartmentalizing into the vacuolar organelle, or excluding to the apoplast and keeping low ion content in the cytosol [18]. Although salinity is a multigenic trait and is governed by the coordinated action of several genes, but previous studies have also supported the efficacy of salinity tolerance imparted by overexpressing single gene either in a model plant or crop plants [25, 27, 30, 32, 34]. The importance of vacuolar Na^+^ compartmentalization in plant salt tolerance has been demonstrated in transgenic plants overexpressing the *NHX1* gene [31, 33]. Functional validation *PgNHX1* in rice revealed that transgenic plants can withstand NaCl up to 100 mM and perform better in terms of seed germination and seed setting [57]. NHX1 antiporter also controls various cellular processes that are not directly linked to salt tolerance. Mutational studies in *Arabidopsis* revealed that NHX1 mutants suffered from numerous developmental abnormalities such as altered cell structure, cell growth, protein processing and vesicular trafficking leading to abnormal phenotype in normal growth environments [58–59]. Taken together, these findings implicate the pivotal role of the antiporter (NHX family) in salt tolerance and supporting the feasibility of generating transgenic plants using Na^+^/H^+^ genes for enhanced salt tolerance.

*In planta* method is a tissue culture-independent genetic transformation carried out using *Agrobacterium tumefaciens* to obtain transformed plants [60]. In the present study, transgenic cumin plants overexpressing the *SbNHX1* gene were developed by an *in planta* transformation protocol. The method is devoid of conventional tissue culture procedures such as callus culture and plant regeneration through indirect pathways because cumin seeds were directly used for transformation with *Agrobacterium* and subsequently transferred to soil for plant development (**Figs 3 and 4**). In the present scenario, the method is widely accepted for transforming crops plants because of several advantages such as less time consuming, reduce labor cost and free of somaclonal variations and other deformities related to plant regeneration [51–52]. Therefore, based on previous findings, an attempt was carried out to perform *Agrobacterium*-mediated *in planta* transformation in cumin using seeds.

In the optimization of the *in planta* transformation protocol in cumin, the results indicated that basal medium for seed germination, pre-culture of seeds and vacuum infiltration were key determinants for increasing the chance of T-DNA transfer into the host plant. The seed vigor is an important factor for successful *in planta* transformation method. Rapidly dividing cells of germinating seeds could be easily targeted for *Agrobacterium* infection. For this, the germination response of cumin seeds on different basal media was checked before used for infection with *Agrobacterium* (**Fig 1**). The composition of the nutrient media was found to influence the growth, and full strength MS basal medium was found to be best suitable for seed inoculation in which 4.4 times higher seed emergence was achieved as compared with water agar medium. Seeds inoculated on MS basal medium showed rapid germination leading to the emergence and healthy growth of seedling as compared to other basal media. Moreover rapidly dividing cells could readily promote T-DNA transfer and integration into the host genome. Subramanyam et al. [52] have also reported that the percentage of seed germination is a critical factor for successful *in planta* transformation using seeds as explant.

The competency for *Agrobacterium* infection could be enhanced by pre-culturing the explants to be used for transformation procedure. Growing the seeds in the basal medium could break the dormancy and also promote mobilization of reserved nutrient material needed for germination [61]. Thus, transformation efficiency could be increased significantly because actively dividing cells were known to be more susceptible to agro-infection leading to T-DNA delivery and integration [52, 62]. In this study, the duration of pre-culturing of seeds before infection with *Agrobacterium* was found to be crucial for enhancing the transformation efficiency. Seeds were kept in dark incubation for a different time interval before infection with *Agrobacterium* to identify the precise stage at which maximum transformation could be achieved (**Fig 1**). The highest number of GUS spots were observed in 7 days old germinated seeds which was 16.6 times higher than five days old germinated seed used for transformation. However as the duration of pre-culture was increased beyond seven days, the transformation frequency was drastically reduced. The GUS spots were uniformly distributed over the different regions germinated seedling covering the cotyledonary leaf, hypocotyl, and radicle in 7 days old pre-cultured seed used for *Agrobacterium* infection. However, the subsequent increase in pre-culture duration of seed to 9 and 11 days resulted in drastic reduction in visible blue foci which was found to be restricted to the junction point of hypocotyl and radicular part of germinated seedling. Furthermore, seven days old germinated seeds were easy to handle for transformation procedure as compared to 9 and 10 days old germinated seeds. Results are in the agreement with previous studies demonstrating the role of actively dividing cells in increasing the transformation efficiency. Braun [63] have emphasized that actively dividing plant cells were in a state of de-nova meristematic activity, resulting in wound-induced reaction necessary for attachment of *Agrobacterium* to plant cell surface for T-DNA transfer. Also, these cells were found to be expressing a higher level of cellular totipotency with a low degree of physiological and morphological specializations, and can revert to cellular dedifferentiation easily from the quiescent stage [62].

Vacuum infiltration is now routinely used for transformation method for enhancing the accessibility of *Agrobacterium* to penetrate deeper into the plant cells for infection [64]. Hence in the present study, seven days pre-cultured seeds were submerged in *Agrobacterium* suspension, and vacuum was applied for 10 minutes to facilitate the *Agrobacterium* cells to enter the plant tissue (**Fig 2**). In the developing embryos of seeds, various primordia develop, but only the embryonic apical meristems were reported to produce shoot apex would produce the germ cells, *i*.*e*. eggs and pollen [39]. So very few piercing were created in the germinated cumin seeds using a fine needle to prevent damaging effect of injury on plumules that will, later on, form shoots. Lin et al. [39] also observed that plumules of the seeds fail to develop shoots due to the direct piercing of needle into the plumules. So after creating injury by fine needle, the vacuum was applied that assisted deeper penetration of the suspension of *Agrobacterium* to enter into the seed of cumin. The region expressing blue loci on transformed seedlings was found to increase dramatically and showed 2.2 times higher GUS spots in vacuum treated as compared to seedlings with no vacuum treatment. Also, the GUS spots were uniformly distributed along the seedling as compared to control. The transformation efficiency was approximately 82% for vacuum treatment seed whereas transformation efficiency remained about 36% in control when no vacuum was used. The application of vacuum infiltration has been reported to enhance transformation efficiency in several crop species generated by *in planta* transformation such as rice [39], brassica [65] and sugarcane [51].

After studying the key components such as basal media for seed germination, pre-culture of seeds and vacuum infiltration, *Agrobacterium*-mediated *in planta* transformation of cumin seeds were carried out. The percentage of explants that survived the process of inoculation and attained maturity was 8.5%. A total of 11 surviving plants at flowering stage were used for confirmation of putative transgenic lines by PCR amplification using *SbNHX1*, *uidA* and *hptII* gene specific primers, out of which 9 transgenic lines were found to be positive that gave amplification of 172 bp, 1280 bp and 963 bp, respectively (**Fig 5**).

The Southern hybridization demonstrated the copy number of *SbNHX1* gene integrated into the genome of transgenic cumin lines but not in WT plants (**Fig 5**). The study revealed a single copy insertion in line L3 and L5. However, double copy gene integration was observed in L10 and L13 transgenic lines. The RT-PCR using *actin* and *SbNHX1* showed the overexpression of *SbNHX1*in transgenic lines but not in WT plant (**Fig 5**). The histochemical GUS staining showed intense visible blue spots while it was absent in WT (**Fig 3**). The accumulation of insoluble indolyl dye in the transformed tissue is mainly because of activity of the *β*-glucosidase enzyme on its substrate 5-bromo-4-chloro-3-indolyl-*β*-D-glucuronic acid, cyclohexylammonium salt (X-Gluc) [66]. The *gus* present in the gene construct encodes for this enzyme indicates expression of functionally active proteins from the transgenes.

To determine the efficacy of *SbNHX1* transgene integrated into the genome of any plant, the proper monitoring of the physiological status under stress condition is crucial. The physiological analysis of transgenic lines of cumin was performed by subjecting these plants to salt treatment for 15 days at 200 mM NaCl stress (**Fig 6**). Various physio-biochemical processes get disturbed under salinity stress, and the severity of damage can be monitored by measuring the level of electrolyte leakage, MDA, proline and photosynthetic pigments during stress. These are considered as notable indicators for the physiological status of the plant [33]. It was observed that transgenic lines carrying the *SbNHX1* gene (L3, L5, L10 and L11) performed better and maintained healthy growth as compared to WT plants at 200 mM NaCl salt stress by observing the physio-biochemical status.

The electrolyte leakage and lipid peroxidation are common phenomena due to the membrane damage caused by the generation of reactive oxygen species (ROS) as a consequence of salinity stress in plants. The electrolyte leakage is mainly due to activation of cation channels resulting in the efflux of K^+^ and its counter ions Cl^-^, NO^3-^, cittrate^3-^ and malate^2-^ [67] and production of malondialdehyde is caused by lipid peroxidation [68]. The present study exhibited lower electrolyte leakage and MDA content in transgenic cumin lines with respect to WT plants under salt stress (200 mM). Transgenic castor performed better and showed enhanced salt tolerance due to ectopic expression of the *SbNHX1* gene in terms of MDA and electrolyte leakage as compared to WT plants [33].

Proline is an organic osmolyte, known to accumulate during salt stress in a variety of plants to provide osmotic balance and protection to cellular enzymes [18, 33]. During salt treatment (200 mM NaCl), the proline content was increased in both WT and transgenic plants. However, the transgenic lines maintained lower proline content as compared with that of wild type. It justifies that transgenic cumin performed better under salinity stress due to the expression of *SbNHX1* gene. The higher content of various photosynthetic pigments (Chl a, Chl b, Chl a+b and carotenoid) in transgenic plants as compared to WT demonstrated the lower deterioration of chlorophyll, representing the better tolerance ability to 200 mM salinity stress. Several other studies have also shown that protective role of the vacuolar *SbNHX1* gene in ameliorating the Na^+^ toxicity. Transgenic jatropha and castor overexpressing *SbNHX1* gene showed better growth and chlorophyll amount when subjected to a different level of salinity stress as compared to non-transformed plants [31, 33]. Thus, the above results confirmed that overexpression of the *SbNHX1* gene in transgenic cumin supported the maintenance of better physio-biochemical status and showed salt endurance compared to WT plants at 200 mM NaCl salt in laboratory conditions.

## Conclusion

In the study, an efficient tissue culture-independent *in planta* genetic transformation method was established in cumin for the first time. Transgenic cumin plants were developed using *SbNHX1* gene that encodes for an antiporter (Na^+^ and H^+^) to ascertain the optimized method. Transgenic cumin plants were thriving well under salinity stress (200 mM NaCl) compared to WT plants as evident by higher photosynthetic pigments and lower physio-biochemical indicators (electrolyte leakage, proline, and malondialdehyde). Transgenic cumin plants overexpressing *SbNHX1*gene showed adequate tolerance under salinity stress and thus could be used for the cultivation in salt affected areas for the sustainable agriculture.

## References

[1] Kaefer CM, Milner JA. The role of herbs and spices in cancer prevention. J. Nutr. Biochem. 2008; 19: 347–361. 10.1016/j.jnutbio.2007.11.003 18499033 PMC2771684

[2] Kabak B, Dobson AD. Mycotoxins in Spices and Herbs: An Update. Crit. Rev. Food Sci. Nutr. 2015; 10.1080/10408398.2013.772891 26528824

[3] Johri RK. *Cuminum cyminum* and Carum carvi: An update. Pharmacogn Rev. 2011; 5: 63 10.4103/0973-7847.79101 22096320 PMC3210012

[4] Sowbhagya HB. Chemistry, technology, and nutraceutical functions of cumin (*Cuminum cyminum* L): An overview. Crit. Rev. Food Sci. Nutr. 2013; 53: 1–10. 10.1080/10408398.2010.500223 23035918

[5] Pandey S, Patel MK, Mishra A, Jha B. Physio-Biochemical composition and untargeted metabolomics of cumin (*Cuminum cyminum* L.) make it promising functional food and help in mitigating slinity stress. PloS One. 2015; 10: e0144469 10.1371/journal.pone.0144469 26641494 PMC4671573

[6] Rebey IB, Zakhama N, Karoui IJ, Marzouk B. Polyphenol composition and antioxidant activity of cumin (*Cuminum cyminum* L.) seed extract under drought. J. Food Sci. 2012; 77: 734–739. 10.1111/j.1750-3841.2012.02731.x 22671525

[7] Singh N, Mishra A, Joshi M, Jha B. Microprojectile bombardment mediated genetic transformation of embryo axes and plant regeneration in cumin (*Cuminum cyminum* L.). Plant Cell Tiss. Org. 2010; 103: 1–6.

[8] Wei J, Zhang X, Bi Y, Miao R, Zhang Z, Su H. Anti-inflammatory effects of cumin essential oil by blocking JNK, ERK, and NF-κB signaling pathways in LPS-stimulated RAW 264.7 Cells. Evidence-Based Complement. Alternat. Med. 2015; 2015: Article ID 474509. 10.1155/2015/474509 PMC4575746 26425131

[9] Morshedi D, Aliakbari F, Tayaranian‐Marvian A, Fassihi A, Pan‐Montojo F, Pérez‐Sánchez H. Cuminaldehyde as the major component of *Cuminum cyminum*, a natural aldehyde with inhibitory effect on alpha‐synuclein fibrillation and cytotoxicity. J. Food Sci. 2015; 80: 2336–2345. 10.1111/1750-3841.13016 26351865

[10] Ebrahimie E, Habashy AA, Mohammadie-Dehcheshmeh M, Ghannadha MR, Ghareyazie B, Yazdi-Amadi B. Direct shoot regeneration from mature embryo as a rapid and genotype-independent pathway in tissue culture of heterogeneous diverse sets of cumin (*Cuminum cyminum* L.) genotypes. In Vitro Cell Dev. Biol. Plant. 2006; 42: 455–460.

[11] Rengasamy P. Transient salinity and subsoil constraints to dryland farming in Australian sodic soils: an overview. Anim. Prod. Sci. 2002; 42: 351–361.

[12] Lal R, Stewart BA. (Eds.). World soil resources and food security. CRC Press; 2011.

[13] Koocheki A, Mehdi NM, Azizi G. Effect of drought, salinity, and defoliation on growth characteristics of some medicinal plants of Iran. J. Herbs Spices Med. Plants. 2008; 14:137–153

[14] Shao HB, Chu LY, Jaleel CA, Zhao CX. Water-deficit stress-induced anatomical changes in higher plants. Comptes Rendus Biologies. 2008; 331: 215–225. 10.1016/j.crvi.2008.01.002 18280987

[15] Hassanzadehdelouei M, Vazin F, Nadaf J. Effect of salt stress in different stages of growth on qualitative and quantitative characteristics of cumin (*Cuminum cyminum* L.). Cercetari Agro. Moldova. 2013; 46: 89–97.

[16] Shoor M, Afrousheh M, Rabeie J, Vahidi M. The effect of salinity priming on germination and growth stage of Cumin (*Cuminum cyminum* L). Research Journal of Agriculture and Environmental Management. 2014; 3: 340–352.

[17] Qadir M, Quillérou E, Nangia V, Murtaza G, Singh M, Thomas RJ et al Economics of salt-induced land degradation and restoration. Nat. Resour. Forum. 2014; 38: 282–295.

[18] Pirasteh-Anosheh H, Ranjbar G, Pakniyat H, Emam Y. Physiological mechanisms of salt stress tolerance in plants, in Plant-Environment Interaction: Responses and Approaches to Mitigate Stress (eds Azooz M. M. and Ahmad P.), John Wiley & Sons, Ltd, Chichester, UK; 2016.

[19] Meena RS. Characterization and evaluation of cumin (*Cuminum cyminum* L.) germplasm. International J. Seed Spices. 2015; 5: 99–102.

[20] Bairwa RK, Solanki RK, Sharma YK, Meena RS. Phenotypic variability in cumin (*Cuminum cyminum* L.) for important agro-morphological traits. International J. Seed Spices. 2015; 5: 68–70.

[21] Hunault G, Desmarest P, Manoir JD. Foeniculum vulgare Miller: cell culture. Regeneration and the production of anethole, in Biotechnology in agriculture and forestry: medicinal and aromatic plants II (ed Y. P. S. Bajaj), 1989. pp. 185–212.

[22] Bahraminejad A, Mohammadi-Nejad G, Kadir MA, Yusop MRB, Samia MA. Molecular diversity of Cumin (*Cuminum cyminum* L.) using RAPD markers. Aust. J. Crop Sci. 2012; 6: 194.

[23] Pandey S, Mishra A, Patel MK, Jha B. An efficient method for Agrobacterium-mediated genetic transformation and plant regeneration in cumin (*Cuminum cyminum* L.). Appl. Biochem. Biotech. 2013; 171: 1–9. 10.1007/s12010-013-0349-1 23813408

[24] Mishra A, Tomar A, Bansal S, Khanna VK, Garg GK. Temporal and spatial expression analysis of gamma kafirin promoter from Sorghum (*Sorghum bicolor* L. Moench) var. M 35–1. Mol. Biol. Rep. 2008; 35: 81–88. 17273894 10.1007/s11033-007-9056-8

[25] Jha B, Sharma A, Mishra A. Expression of *SbGSTU* (*tau class glutathione S-transferase*) gene isolated from *Salicornia brachiata* in tobacco for salt tolerance. Mol. Biol. Rep. 2011; 38: 4823–4832. 10.1007/s11033-010-0625-x 21136169

[26] Chaturvedi AK, Patel MK, Mishra A, Tiwari V, Jha B. The *SbMT-2* gene from a halophyte confers abiotic stress tolerance and modulates ROS scavenging in transgenic tobacco. PloS One. 2014; 9: e111379 10.1371/journal.pone.0111379 25340650 PMC4207811

[27] Singh N, Mishra A, Jha B. Over-expression of the peroxisomal ascorbate peroxidase (*SbpAPX*) gene cloned from halophyte *Salicornia brachiata* confers salt and drought stress tolerance in transgenic tobacco. Mar. Biotechnol. 2014; 16: 321–332. 10.1007/s10126-013-9548-6 24197564

[28] Tiwari V, Chaturvedi AK, Mishra A, Jha B. The transcriptional regulatory mechanism of the peroxisomal ascorbate peroxidase (*pAPX*) gene cloned from an extreme halophyte, *Salicornia brachiata*. Plant Cell Physiol. 2014; 55: 201–217. 10.1093/pcp/pct172 24285755

[29] Tiwari V, Patel MK, Chaturvedi AK, Mishra A, Jha B. Functional Characterization of the *Tau class glutathione-s-transferases* gene (*SbGSTU*) promoter of *Salicornia brachiata* under salinity and osmotic stress. PloS One. 2016; 11: e0148494 10.1371/journal.pone.0148494 26885663 PMC4757536

[30] Udawat P, Jha RK, Sinha D, Mishra A and Jha B. Overexpression of a Cytosolic Abiotic Stress Responsive Universal Stress Protein (*SbUSP*) Mitigates Salt and Osmotic Stress in Transgenic Tobacco Plants. Front. Plant Sci. 2016; 7:518 10.3389/fpls.2016.00518 27148338 PMC4838607

[31] Joshi M, Jha A, Mishra A, Jha B. Developing transgenic Jatropha using the *SbNHX1* gene from an extreme halophyte for cultivation in saline wasteland. PloS One. 2013; 8: e71136 10.1371/journal.pone.0071136 23940703 PMC3733712

[32] Singh N, Mishra A, Jha B. Ectopic over-expression of peroxisomal ascorbate peroxidase (*SbpAPX*) gene confers salt stress tolerance in transgenic peanut (*Arachis hypogaea*). Gene. 2014; 547: 119–125. 10.1016/j.gene.2014.06.037 24954532

[33] Patel MK, Joshi M, Mishra A, Jha B. Ectopic expression of *SbNHX1* gene in transgenic castor (*Ricinus communis* L.) enhances salt stress by modulating physiological process. Plant Cell Tiss. Org. 2015; 122:1–14.

[34] Tiwari V, Chaturvedi AK, Mishra A, Jha B. Introgression of the *SbASR-1* gene cloned from a halophyte *Salicornia brachiata* enhances salinity and drought endurance in transgenic groundnut (*Arachis hypogaea*) and acts as a transcription factor. PloS One. 2015; 10: e0131567 10.1371/journal.pone.0131567 26158616 PMC4497679

[35] Barampuram S, Zhang ZJ. Recent advances in plant transformation. In Birchler JA (ed.), Method Mol. Biol. 2011; 701:1–35. 10.1007/978-1-61737-957-4_1 21181522

[36] Joshi M, Mishra A, Jha B. Efficient genetic transformation of *Jatropha curcas* L. by microprojectile bombardment using embryo axes. Ind. Crop Prod. 2011; 33: 67–77.

[37] Joshi M, Mishra A, Jha B. NaCl plays a key role for *in vitro* micropropagation of *Salicornia brachiata*, an extreme halophyte. Ind. Crop Prod. 2012; 35: 313–316.

[38] Tiwari V, Chaturvedi AK, Mishra A, Jha B. An efficient method of *Agrobacterium*-mediated genetic transformation and regeneration in local Indian cultivar of groundnut (*Arachis hypogaea*) using grafting. Appl. Biochem. Biotech. 2015; 175: 436–453. 10.1007/s12010-014-1286-3 25308617

[39] Lin J, Zhou B, Yang Y, Mei J, Zhao X, Guo X, Hang X, Tang D, Lin X. Piercing and vacuum infiltration of mature embryo: a simplified method for *Agrobacterium*-mediated transformation of indica rice. Plant Cell. Rep. 2009; 28: 1065–1074. 10.1007/s00299-009-0706-2 19455339

[40] Larkin PJ, Scowcroft WR. Somaclonal variation–a novel source of variability from cell cultures for plant improvement. Theor. Appl. Genet. 1981; 60: 197–214. 10.1007/BF02342540 24276737

[41] Evans DA end Sharp WR. Application of somaclonal variations. Nat. Biotechnol. 1986; 4, 528–532.

[42] Bairu MW, Aremu AO, Van Staden J. Somaclonal variation in plants: causes and detection methods. Plant Growth Regul. 2011; 63(2), 147–173.

[43] Rao KS, Sreevathsa R, Sharma PD, Keshamma E, Kumar MU. *In planta* transformation of pigeon pea: a method to overcome recalcitrancy of the crop to regeneration *in vitro*. Physiol. Mol. Biol. Plants. 2008; 14(4), 321–328. 10.1007/s12298-008-0030-2 23572898 PMC3550634

[44] Lutts S, Kinet JM, Bouharmont J. NaCl-induced senescence in leaves of rice (*Oryza sativa* L.) cultivars differing in salinity resistance. Ann. Bot. 1996; 78: 389–398.

[45] Adams P, Thomas JC, Vernon DM, Bohnert HJ, Jensen RG. Distinct cellular and organismic responses to salt stress. Plant Cell Physiol. 1992; 33: 1215–1223.

[46] Padan E, Schuldiner S. Molecular physiology of the Na^+^/H^+^ antiporter in *Escherichia coli*. J. Exp. Biol. 1994; 196: 443–456. 7823039 10.1242/jeb.196.1.443

[47] Jha B, Singh NP, Mishra A. Proteome profiling of seed storage proteins reveals the nutritional potential of *Salicornia brachiata* Roxb., an extreme halophyte. J. Agric. Food Chem. 2012; 60: 4320–4326. 10.1021/jf203632v 22494338

[48] Mishra A, Joshi M, Jha B. Oligosaccharide mass profiling of nutritionally important *Salicornia brachiata*, an extreme halophyte. Carbohydr. Polym. 2013; 92: 1942–1945. 10.1016/j.carbpol.2012.11.055 23399241

[49] Mishra A, Patel MK, Jha B. Non-targeted metabolomics and scavenging activity of reactive oxygen species reveal the potential of *Salicornia brachiata* as a functional food. J. Funct. Foods. 2015; 13: 21–31.

[50] Park BJ, Liu Z, Kanno A, Kameya T. Transformation of radish (*Raphanus sativus* L.) via sonication and vacuum infiltration of germinated seeds with *Agrobacterium* harboring a group 3 LEA gene from *B*. *napus*. Plant Cell Rep. 2005; 24: 494–500. 15843933 10.1007/s00299-005-0973-5

[51] Mayavan S, Subramanyam K, Arun M, Rajesh M, Dev GK, Sivanandhan G, et al *Agrobacterium* tumefaciens-mediated *in planta* seed transformation strategy in sugarcane. Plant Cell Rep. 2013; 32: 1557–1574. 10.1007/s00299-013-1467-5 23749098

[52] Subramanyam K, Rajesh M, Jaganath B, Vasuki A, Theboral J, Elayaraja D, et al Assessment of factors influencing the *Agrobacterium*-mediated *in planta* seed transformation of brinjal (*Solanum melongena* L.). Appl. Biochem. Biotechnol. 2013; 171: 450–468. 10.1007/s12010-013-0359-z 23852797

[53] Bates LS, Waldern R, Teare ID. Rapid determination of free proline for water stress studies. Plant Soil. 1973; 39:205–207.

[54] Hodges DM, Delong JM., Forney CF, Prange RK. Improving the thiobarbituric acid reactive substances assay for estimating lipid peroxidation in plant tissues containing anthocyanin and other interfering compounds. Planta. 1999; 207: 604–611. 10.1007/s00425-017-2699-3 28456836

[55] Inskeep WP, Bloom PR. Extinction coefficients of chlorophyll a and b in N, N-dimethylformamide and 80% acetone. Plant Physiol. 1985; 77: 483–485. 16664080 10.1104/pp.77.2.483 PMC1064541

[56] Porra RJ, Thompson WA, Kriedemann PE. Determination of accurate extinction coefficients and simultaneous equations for assaying chlorophylls a and b extracted with four different solvents: verification of the concentration of chlorophyll standards by atomic absorption spectroscopy. Biochim. Biophys. Acta. 1989; 975: 384–394.

[57] Verma D, Singla-Pareek SL, Rajagopal D, Reddy MK, Sopory SK. Functional validation of a novel isoform of Na^+^/H^+^ antiporter from *Pennisetum glaucum* for enhancing salinity tolerance in rice. J. Biosci. 2007; 32: 621–628. 17536181 10.1007/s12038-007-0061-9

[58] Sottosanto JB, Gelli A, Blumwald E. DNA array analyses of *Arabidopsis thaliana* lacking a vacuolar Na^+^/H^+^ antiporter: impact of *AtNHX1* on gene expression. Plant J. 2004; 40: 752–771. 15546358 10.1111/j.1365-313X.2004.02253.x

[59] Sottosanto JB, Saranga Y, Blumwald E. Impact of *AtNHX1*, a vacuolar Na^+^/H^+^ antiporter, upon gene expression during short-and long-term salt stress in *Arabidopsis thaliana*. BMC Plant Biol. 2007; 7: 18 17411438 10.1186/1471-2229-7-18 PMC1853094

[60] Feldmann KA, Marks MD. *Agrobacterium*-mediated transformation of germinating seeds of *Arabidopsis thaliana*: a non-tissue culture approach. Mol. Gen. Genet. 1987; 208: 1–9.

[61] Gordon-Kamm W, Dilkes BP, Lowe K, Hoerster G, Sun X, Ross M, et al Stimulation of the cell cycle and maize transformation by disruption of the plant retinoblastoma pathway. Proc. Natl. Acad. Sci. 2002; 99: 11975–11980. 12185243 10.1073/pnas.142409899 PMC129379

[62] Sangwan RS, Bourgeois Y, Brown S, Vasseur G, Sangwan-Norreel B. Characterization of competent cells and early events of *Agrobacterium*-mediated genetic transformation in *Arabidopsis thaliana*. Planta. 1992; 188: 439–456. 10.1007/BF00192812 24178335

[63] Braun AC. Conditioning of the host cell as a factor in the transformation process in crown gall. Growth 1952; 16: 65–74. 12980380

[64] Bechtold N, Ellis J, Pelletier G. *In planta Agrobacterium* mediated gene transfer by infiltration of adult *Arabidopsis thaliana* plants. CR Acad. Sci. Ser. III Sci. Vie. 1993; 316: 1194–1199.

[65] Bai J, Wu F, Mao Y, He Y. *In planta* transformation of *Brassica rapa* and *B*. *napus* via vernalization-infiltration methods. Protoc. Exch. 2013; 10: 1028.

[66] Jefferson RA, Kavanagh TA, Bevan MW. GUS fusions: beta-glucuronidase as a sensitive and versatile gene fusion marker in higher plants. EMBO J. 1987; 6: 3901 3327686 10.1002/j.1460-2075.1987.tb02730.x PMC553867

[67] Demidchik V, Straltsova D, Medvedev SS, Pozhvanov GA, Sokolik A, Yurin V. Stress-induced electrolyte leakage: the role of K^+^-permeable channels and involvement in programmed cell death and metabolic adjustment. J. Exp. Bot. 2014; 65: 1259–1270. 10.1093/jxb/eru004 24520019

[68] Moore K, Roberts LJ. Measurement of lipid peroxidation. Free Radical Res. 1998; 28: 659–671. 9736317 10.3109/10715769809065821

